# Musculoskeletal Disorders and Quality of Life in Chilean Teachers: A Cross-Sectional Study

**DOI:** 10.3389/fpubh.2022.810036

**Published:** 2022-03-29

**Authors:** Gustavo Vega-Fernández, Enrique Olave, Pablo A. Lizana

**Affiliations:** ^1^Laboratory of Epidemiology and Morphological Sciences, Instituto de Biología, Pontificia Universidad Católica de Valparaíso, Valparaíso, Chile; ^2^Programa de Magister en Ciencias Mención Morfología, Universidad de La Frontera, Temuco, Chile; ^3^Facultad de Medicina, Universidad de La Frontera, Temuco, Chile

**Keywords:** mental health, physical health, quality of life, school teachers, gender, musculoskeletal disorders

## Abstract

**Introduction:**

Teaching professionals have reported multiple conditions associated with low quality of life (QoL) perception. Various studies have also reported high prevalence of musculoskeletal disorders (MSD). In this context, there are few studies regarding the association between low QoL perception and MSD suffering in teachers.

**Objective:**

Therefore, in this study the aim was evaluate MSD prevalence and its association with teachers' QoL perception.

**Participants and Methods:**

A total sample of 544 Chilean teachers were included in a cross-sectional study. MSD prevalence was evaluated via the Standardized Nordic Questionnaire and QoL perception via the Short-Form 36 Health Survey Instrument. Multinomial logistic regression was applied to evaluate the association between MSD and QoL perception adjusted for gender and age.

**Results:**

A total of 91% of teachers have had some MSD in the last 12 months, and 28.86% have had 6 or more painful regions. Females showed greater MSD prevalence than males. Teachers who reported no MSD had higher QoL scores compared to teachers with MSD. The group of teachers with the most MSD (≥p75) saw significant increases in the risk of having low scores in the physical (OR: 2.82) and mental components (OR: 2.65) of QoL. By contrast, teachers without MSD have a buffer effect for their QoL (physical OR: 0.2; mental OR: 0.44).

**Conclusions:**

High MSD prevalence suggest that preventive and informative actions must be taken regarding these disorders to protect teachers' mental and physical health, considering the multiple risk factors to which teachers are exposed given their work conditions in Chile and worldwide.

## Introduction

Musculoskeletal disorders (MSD) are musculoskeletal system pathologies which produce pain and are sometimes accompanied by mobility limitations (1, 2). They are also some of the most prevalent public health problems among the working population and the general global population (1), representing one of the main causes of work absenteeism (3) and causing major economic costs for national health systems worldwide (4–6). Regarding physical health, MSD have been associated with workplace ergonomics and infrastructure (7), chronic health conditions including obesity and type 2 diabetes (8), repetitive movements, bad posture, lifting heavy objects and general physical stress (9–11). In mental health, MSD have been associated with high psychosocial demands, high mental workplace stress and workplace harassment (12–15), and feelings of depression, anxiety, and job dissatisfaction (10, 16, 17). All of these health conditions significantly im-pact the Quality of Life (QoL) of working groups and the general population (18, 19).

The teaching sector has reported various occupational ailments due to high physical and mental demands (20–22), along with various diseases related to MSD (23, 24). Worldwide MSD reports among teachers indicate that these symptoms' prevalence fluctuates between 30 and 90% in this work group (24) and are related with various sociodemographic factors and certain health conditions among teachers (25–27). For example, some studies report high prevalence of pain in the lower back, neck and shoulders associated with depressive conditions, anxiety, and high psychosocial demands at work (25, 28), and high foot pain prevalence associated with age, body mass index (BMI), inappropriate footwear and long hours standing (29), along with teachers with high lower back pain rates associated with sleep disorders, irregular physical activity and staying on their feet at work (30).

MSD are also associated with low QoL perception in various working groups worldwide (31, 32), as well as in general population studies (33, 34). Among teachers, those suffering from MSD have also reported low QoL scores, impacting their physical and mental health (20, 35, 36).

Chilean teachers have reported that some chronic health conditions generate significant QoL impact, especially on the Mental Component Summary (20). However, reports associating MSD with QoL in Chilean teachers are scarce. Therefore, the objective of this study is to determine MSD prevalence among teachers and its association with QoL mental and physical components.

## Materials and Methods

### Participants

The target population was all Chilean teachers [*N* = 2,49,865; (37)]. To calculate the sample size, the variable with the greatest variance for this group was selected according to the literature published. The sample was determined with the variable MSD for Chilean workers [49.8%; (38)]. The sample was calculated with 95% confidence and a 5% error. In addition, the sample size was increased by 25% due to possible dropouts. Minimum sample was 511.

This cross-sectional study was done among currently active teachers working in various types of schools. The schools were randomly selected. There were 697 initial participants in the study. Of these, 132 did not complete some of the surveys and 21 had missing data. In total, we analyzed 544 teachers from 28 urban schools in three Chilean regions, namely: northern zone: Arica and Parinacota Region (41%); central zone: Valparaíso Region (36%); and southern zone: Araucanía Region (23%). Measurements were applied between October 2018 and October 2019.

### Instruments

#### Musculoskeletal Disorders

To evaluate MSD, we used the Spanish version of the Standardized Nordic Questionnaire adapted and validated for various countries including Chile (39–41). This instrument grants information about teachers' pain prevalence in 12 body areas: neck, right shoulder, left shoulder, right forearm/elbow, left forearm/elbow, right hand/wrist, left hand/wrist, upper back, lower back, hips/buttocks/thighs, knees, feet/ankles. The first part of the questionnaire consisted in detecting whether or not pain was present in any of the 12 previously mentioned areas, within the last 12 months. The second part of the questionnaire investigated whether incapacitating pain happened within any of the 12 body areas in the last 12 months. The instrument uses short answers (yes/no) to prevent self-report from having inaccuracies and subjective biases regarding pain scale and TME durations in painful regions.

### Quality of Life

To evaluate teachers' QoL, we used the Spanish version with syntactic and semantic adjustments for Chilean expressions (42, 43) of the “Short-Form 36 Health Survey Instrument (SF-36)” questionnaire created and standardized in the USA (44). This instrument evaluates QoL in individuals using 36 Likert-form questions, calibrated to obtain a diagnosis in eight health dimensions: physical function, physical role, body pain, general health perception, vitality, social function, emotional role, and mental health. These 8 scales are gathered in two components: Physical Component Summary (PCS) and Mental Component Summary (MCS). The questions for each of the eight dimensions add up to a score on a scale of 0 to 100, which is then standardized via a T-Score value for each scale and PCS and MCS summary measures, calculated according to the standardization indicated by the creator (44). Values greater than T-Score 50 indicate good QoL perception while scores below T-Score 50 indicate poor QoL perception. Regarding the reliability of the scale, the Cronbach's alpha coefficient was α = 0.883 for the physical function scale, α = 0.860 for the limitations due to physical problems scale, α = 0.868 for the bodily pain scale, α = 0.859 for the general health perceptions scale, α = 0.854 for the vitality scale, α = 0.853 for the social functioning scale, α = 0.858 for the role limitations due to emotional problems scale, α = 0.852 for the mental health scale, α = 0.874 for the Physical Component Summary and α = 0.861 for the Mental Component Summary.

### Proceedings

Instruments were previously explained to the teachers by the researchers in person. Teachers also had to sign informed consent indicating their voluntary participation, without payment or incentives in any point of the study. All procedures in this study met with bioethical standards according to the Helsinki Declaration (45) and were approved by the Ethics Committee at Pontificia Universidad de Valparaíso, Chile (n°BIOEPUCV-H 160-2017).

### Statistical Analysis

In statistical analysis, we used STATA MP2 V.16 software for Windows. For associations between categorical variables, the chi-squared test and Fisher's exact test (frequencies below 5) were used. In mean comparisons, the *t*-test was used for parametric variables while the Wilcoxon's test was used for non-parametric variables according to the Shapiro-Wilk normality test. Differences and associations between sociodemographic variables were analyzed according to gender. Two age categories were used (≤44 years old and ≥45 years old) according to the cut-off scores used in the Chilean National Health Survey 2009/2010 (46). MSD prevalence in each region and body segment were described according to gender and age category in the last 12 months without movement limitations and in the last 12 months with movement limitations. We applied ANOVA to evaluate differences between scores on each of the eight QoL dimensions regarding MSD prevalence grouped in 3 categories as a response variable (without symptoms, ≥ p10– <p75 [1 to 5 regions], and ≥p75 [6 or more regions]), followed by a *post-hoc* test (Bonferroni). Finally, two logistic regression models were done with the highest and lowest T-Score 50 scores on the Physical Component Summary and Mental Component Summary QoL measurements as dependent variables, while for independent variables we used the group without pains (0 painful regions), and the group with high pain prevalence (≥p75–6 or more regions without limitations) adjusted for gender and age. The goodness of fit calculation for each model was done with a Hosmer-Lemeshow test (a value above 0.05 indicates that the model fits the data).

## Results

The sociodemographic characteristics of the teachers participating in this study are presented in Table 1, compared by gender. In total, 544 teachers were analyzed, of which 390 were women (72%). Mean age of the total sample was 40.3 ± 12 years; 66% of participants fell in the first age category (≤44 years). There was a significant association (*p* < 0.05) between the female gender and the presence of 4 or more musculoskeletal pains (≥p50) and low scores on the physical component of QoL (<T-Score). In addition, it is observed that younger teachers have significantly greater MSD (>5 regions with pain). On the other hand, a significant association is observed between the high prevalence of MSD and the components of QoL scores (PCS and MCS; see Table 2).

**Table 1:** Sociodemographic characteristics, presence of musculoskeletal disorders and quality of life, evaluated by gender in Chilean teachers.

	**Total sample**	**Gender**		
		**Male (*n* = 154)**	**Female (*n* = 390)**	***P***
**Age (years)^c^**	40.30 ± 12.05	40.12 ± 13.04	40.35 ± 11.66	0.614^a^
≤ 44	359 (66)	107 (69.48)	252 (64.62)	0.315
≥45	185 (34.01)	47 (30.52)	138 (35.38)	
**Marital status**				
Single	238 (43.75)	73 (47.40)	165 (42.31)	0.010^b^
Married/partnered	261 (47.98)	77 (50.00)	184 (47.18)	
DWW	45 (8.27)	4 (2.60)	41 (10.51)	
**Type of school**				
Public (state)	181 (33.27)	45 (29.22)	136 (34.87)	0.196^b^
Private (subsidized)	310 (57)	97 (62.99)	213 (54.62)	
Private	53 (9.7)	12 (7.79)	41 (10.51)	
(non-subsidized)				
**Type of contract**				
Fixed-term^a^	192 (35.69)	48 (31.58)	144 (37.31)	0.212^b^
Indefinite-term^a^	346 (64.31)	104 (68.42)	242 (62.69)	
**Domestic work**				
<15	483 (88.7)	143 (92.86)	340 (87.18)	0.059^b^
≥15	61 (11.21)	11 (7.14)	50 (12.82)	
**MSD^d^**				
< p50^e^	239 (43.93)	81 (56.60)	158 (40.51)	0.01^b^
≥p50^e^	305 (56.10)	73 (47.40)	232 (59.49)	
< p75^f^	387 (71.14)	116 (75.32)	271 (69.49)	0.176^b^
≥p75^f^	157 (28.86)	38 (24.68)	119 (30.51)	
**PCS**
< T-Score	277 (50.92)	67 (43.51)	210 (53.85)	0.030^b^
≥T-Score	267 (49.08)	87 (56.49)	180 (46.15)	
**MCS**
< T-Score	327 (60.11)	85(55.19)	242 (62.05)	0.141^b^
≥T-Score	217 (39.89)	69 (44.81)	148 (38.95)	

a *Wilcoxon test*.

b *Chi-Squared; DWW, Divorced Widow Widower; MSD, Musculoskeletal Disorders; PCS, Physical Component Summary; MCS, Mental Component Summary; T-Score, 50 indicate good QoL perception while scores below 50 indicate poor QoL perception; <15 ≥15, domestic work in hours; P < 0.05*.

c *Data are expressed as mean and standard deviation*.

d *MSD in the last 12 months without limitations*.

e *MSD ≥ 4 regions*.

f *MSD ≥ 6 regions*.

**Table 2:** Sociodemographic characteristics and quality of life, evaluated by the presence of musculoskeletal disorders in Chilean teachers.

	**MSD**					
	**No symptoms**	**With symptoms**	**p-value**	**≤65 painful regions**	**≥painful regions**	***P***
**Age (years)^c^**	40.92 ± 11.48	40.23 ± 12.12	0.48^a^	41.18 ± 12.25	31.12 ± 11.30	<0.01^a^
≤ 44	33 (66.0)	326 (65.99)	0.99^b^	244 (63.05)	115 (73.25)	0.02^b^
≥45	17 (34.00)	168 (34.01)		143 (36.95)	42 (26.75)	
**Gender**
Male	19 (38.00)	135 (27.00)	0.11^b^	116 (29.97)	38 (24.20)	0.117^b^
Female	31 (62.00)	359 (72.67)		271 (70.03)	119 (75.80)	
**Marital status**
Single	15 (30.00)	223 (45.14)	0.108^b^	167 (43.15)	71 (45.22)	0.88^b^
Married/partnered	29 (58.00)	232 (49.96)		187 (48.32)	74 (47.13)	
DWW	6 (12.00)	39 (7.89)		33 (8.53)	12 (7.64)	
**Tipo de subvención**
Public (state)	11 (22.00)	170 (34.41)	0.102^b^	120 (31.01)	61 (38.85)	0.201^b^
Private (subsidized)	31 (62.00)	279 (56.48)		227 (58.66)	83 (52.87)	
Private (non-subsidized)	8 (4.1)	45 (9.11)		40 (10.34)	13 (8.82)	
**Type of contract**
Fixed-terma	15 (30.00)	177 (36.27)	0.378^b^	130 (33.94)	62 (40.00)	0.184^b^
Indefinite-terma	35 (70.00)	311 (63.73)		253 (66.06)	93 (60.00)	
**Domestic work**
<15	45 (90.00)	438 (88.66)	0.775^b^	350 (90.44)	133 (84.71)	0.055^b^
≥15	5 (10.00)	56 (11.34)		37 (9.65)	24 (15.29)	
**PCS**
< T-Score	8 (16.00)	269 (54.45)	<0.001^b^	170 (43.93)	107 (68.15)	<0.001^b^
≥T-Score	42 (84.00)	225 (45.55)		217 (56.07)	50 (31.85)	
**MCS**
< T-Score	18 (36.00)	309 (62.55)	<0.001^b^	206 (53.23)	121 (77.07)	<0.001^b^
≥T-Score	32 (64.00)	185 (37.45)		181 (46.77)	36 (22.93)	

a *Wilcoxon test*.

b *Chi-Squared; DWW, Divorced Widow Widower; PCS, Physical Component Summary; MCS, Mental Component Summary; T-Score, 50 indicate good QoL perception while scores below 50 indicate poor QoL perception; <15 >15, domestic work in hours; P <0.05*.

c *Data are expressed as mean and standard deviation*.

Table 3 shows MSD prevalence in each body region in the total sample and according to age categories during the previous 12 months (with and without movement limitations). Around 91% reported some musculoskeletal pain in the previous 12 months without limitations. The region with the highest pain prevalence was the lower back at 80.85%, followed by the neck/shoulder segment at 67%. The ≤44 years group also presented greater prevalence of pain in the upper limbs and back, while in the lower limbs the highest prevalence was found among older teachers (≥45 years). Furthermore, when evaluating pain with mobility limitations somewhere within the body, the general prevalence was 75.18% in the total sample, with the most common regions being the neck/shoulders (52.39%) and lower back (50.5%). 64.5% of teachers ≤44 years also mentioned having over 5 painful regions with mobility limitations in the previous 12 months.

**Table 3:** Prevalence of musculoskeletal disorders in the last 12 months with and without movement limitations according to age ranges.

	**Musculoskeletal disorders**					
	**Last 12 months without limitations**			**Last 12 months with limitations**		
	**Total**	**≤44**	**≥45**	**Total**	**≤44**	**≥45**
Neck	311 (47.17)	220 (61.28)	91 (49.19)	244 (44.85)	169 (47.08)	75 (40.54)
Shoulders	241 (44.30)	167 (46.52)	74 (40)	183 (33.64)	127 (35.38)	56 (30.27)
Neck/Shoulders	364 (66.91)	252 (70.19)	112 (60.54)	285 (52.39)	196 (54.60)	89 (48.11)
Elbows	108 (19.85)	63 (17.55)	45 (24.32)	84 (15.44)	48 (13.37)	36 (19.46)
Wrist/Hands	201 (36.95)	141 (39.28)	60 (32.43)	153 (28.13)	108 (30.08)	45 (24.32)
Any upper limb	413 (75.92)	277 (77.16)	136 (73.51)	325 (59.74)	216 (60.17)	109 (58.92)
Upper back	238 (43.75)	168 (46.8)	70 (37.84)	189 (34.74)	133 (37.05)	56 (30.27)
Low back	331 (80.85)	234 (65.18)	97 (52.43)	275 (50.55)	192 (53.48)	83 (44.86)
Any Back	385 (70.77)	269 (74.93)	116 (62.70)	316 (58.09)	221 (61.56)	95 (51.35)
Hips/Thighs	155 (28.49)	102 (28.41)	53 (28.65)	117 (21.51)	75 (20.89)	42 (22.70)
Knees	248 (45.59)	157 (43.73)	91 (49.19)	198 (36.40)	122 (33.98)	76 (41.08)
Ankles/Feet	196 (36.03)	139 (35.93)	67 (36.22)	152 (27.94)	97 (27.02)	55 (29.73)
Any lower limb	365 (67.10)	235 (65.46)	130 (70.27)	286 (52.57)	181 (50.42)	105 (56.76)
Any MSD	494 (90.81)	326 (90.81)	168 (90.81)	409 (75.18)	271 (75.49)	138 (74.59)
≥p50	305 (56.07)	210 (58.50)^a^	95 (51.35)^a^	292 (53.18)	192 (53.48)^c^	100 (54.05)^c^
≥p75	157 (28.86)	115 (32.03)^b^	42 (22.70)^b^	180 (33.09)	124 (64.54)^d^	156(30.27)^d^
Without MSD	50 (9.19)	33 (9.19)	17 (9.19)	135 (24.82)	88 (24.51)	47 (25.41)

*Data are expressed as frequency (percentage). MSD, musculoskeletal disorder*.

a *MSD ≥ 4 regions*.

b *MSD ≥ 6 regions*.

c *MSD ≥ 3 regions*.

d *MSD ≥ 5 regions*.

MSD prevalence in the previous 12 months without mobility limitations and with limitations presented by gender appear in Table 4. Pain prevalence in at least one body region were greater among women, with 92% reporting pain without limitations and 76.92% presenting pain with limitations. The body segments with the highest prevalence without limitations were the upper limbs (78.72%) and neck/shoulders (69.49%) corresponding to women. The only body region with a slightly higher pain prevalence in men was the ankle/foot in both survey segments (with and without limitations). Females had a higher prevalence of presenting 4 painful regions with limitations (36.15%).

**Table 4:** Prevalence of musculoskeletal disorders in the last 12 months with and without movement limitations according to gender.

	**Musculoskeletal disorders**			
	**Last 12 months without limitations**		**Last 12 months with limitations**	
	**Male**	**Female**	**Male**	**Female**
Neck	74 (48.05)	237 (60.77)	55 (35.71)	189 (48.46)
Shoulders	66 (42.86)	165 (44.87)	48 (31.17)	135 (34.62)
Neck/Shoulders	93 (60.39)	271 (69.49)	69 (44.81)	216 (55.38)
Elbows	27 (17.53)	81 (20.77)	20 (12.99)	64 (16.41)
Wrist/Hands	51 (33.12)	150 (38.46)	36 (23.38)	117 (30)
Any upper limb	106 (68.83)	307 (78.72)	78 (50.65)	247 (63.33)
Upper back	55 (35.71)	183 (46.92)	43 (27.92)	146 (37.44)
Low back	81 (52.60)	250 (64.10)	64 (41.56)	211 (54.10)
Any Back	96 (62.34)	289 (74.10)	78 (50.65)	238 (61.03)
Hips/Thighs	38 (24.68)	117 (30)	25 (16.23)	91 (23.59)
Knees	69 (44.81)	179 (45.90)	55 (35.71)	143 (36.67)
Ankles/Feet	56 (36.36)	140 (35.90)	45 (29.22)	107 (27.44)
Any lower limb	102 (66.23)	263 (67.44)	79 (51.30)	207 (53.08)
Any MSD	135 (87.66)	359 (92.05)	109 (70.78)	300 (76.92)
≥p50	73 (47.40)^a^	232 (59.49)^a^	70 (45.45)^c^	222 (56.92)^c^
≥p75	38 (24.68)^b^	119 (30.51)^b^	39 (25.32)^d^	141 (36.15)^d^
Whitout MSD	19 (12.34)	31 (7.95)	45 (29.22)	90 (23.08)

*Data are expressed as frequency (percentage). MSD, musculoskeletal disorder*.

a *MSD ≥ 4 regions*.

b *MSD ≥ 6 regions*.

c *MSD ≥ 3 regions*.

d *MSD ≥ 5 regions*.

Table 5 shows comparisons of scores in each QoL dimension with three MSD prevalence categories. The results show that all teachers who reported no MSD had significantly higher QoL scores (better QoL) than other categories, especially compared with the group of teachers with prevalence above p75 (6 or more regions without limitations and 5 or more with limitations; *p* < 0.05).

**Table 5:** Evaluation of the differences in the scores of the eight quality of life scales between three categories of MSD according to age ranges.

	**Last 12 months**				**Last 12 months with limitations**			
**Quality of life**	**Without pain^**1**^**	**≤5 painful regions^**2**^**	**≥6 painful regions^**3**^**	***P* ^**(posthoc)**^**	**Without pain^**1**^**	**≤4 painful regions^**2**^**	**≥5 painful regions^**3**^**	***P* ^**(posthoc)**^**
**≤44**
Physical function	53.86 ± 8.99	53.137 ± 5.56	52.022 ± 6.06	0.002^a(c)^	53.57 ± 8.08	53.21 ± 5.11	51.91 ± 5.49	<0.001^a(c)^
Physical problems^e^	52.53 ± 4.48	50.43 ± 5.63	48.10 ± 6.75	<0.001^a(c)^	50.71 ± 5.95	50.44 ± 5.87	48.6131 ± 6.20	0.006^a(d)^
Bodily pain	55.40 ± 5.89	45.49 ± 9.23	40.14 ± 8.26	<0.001^a(c)^	49.90 ± 8.81	44.68 ± 9.44	41.01 ± 8.68	<0.001^a(c)^
General health perceptions	54.31 ± 8.12	47.85 ± 9.31	42.52 ± 10.32	<0.001^a(c)^	50.73 ± 9.079	47.79 ± 9.60	42.65 ± 10.03	<0.001^a(c)^
Vitality	52.05 ± 9.14	47.79 ± 8.57	43.25 ± 8.85	<0.001^b(c)^	50.15 ± 9.54	47.13 ± 8.65	43.79 ± 8.37	<0.001^b(c)^
Social functioning	47.85 ± 9.62	43.44 ± 10.44	39.60 ± 10.68	<0.001^a(c)^	46.60 ± 10.14	43.95 ± 10.45	39.90 ± 10.67	<0.001^a(c)^
Emotional problems^f^	50.69 ± 6.29	48.66 ± 6.51	45.35 ± 7.25	<0.001^a(c)^	49.41 ± 6.32	48.58 ± 6.91	45.69 ± 6.97	<0.001^a(d)^
Mental health	51.43 ± 10.01	47.15 ± 10.02	42.19 ± 9.61	<0.001^a(c)^	48.73 ± 10.66	47.12 ± 9.73	42.61 ± 9.77	<0.001^a(d)^
**≥45**
Physical function	51.25 ± 7.00	49.25 ± 7.00	45.23 ± 8.83	0.003^a(d)^	49.47 ± 7.38	49.77 ± 7.25	45.81 ± 7.87	<0.001^a(d)^
Physical problems^e^	52.30 ± 4.78	50.49 ± 5.18	48.34 ± 6.18	0.015^a(c)^	51.63 ± 4.57	51.01 ± 5.10	47.70 ± 5.96	<0.001^a(d)^
Bodily pain	52.91 ± 9.14	44.80 ± 9.08	38.39 ± 8.57	<0.001^a(c)^	50.37 ± 8.38	44.76 ± 9.02	37.66 ± 7.85	<0.001^a(c)^
General health perceptions	51.24 ± 10.93	49.02 ± 8.14	42.41 ± 10.14	<0.001^a(c)^	50.85 ± 8.68	49.45 ± 7.85	42.57 ± 9.88	<0.001^a(d)^
Vitality	54.75 ± 7.41	49.91 ± 8.62	47.91 ± 7.04	0.011^a(c)^	52.54 ± 7.89	51.24 ± 7.35	45.72 ± 8.59	<0.001^a(d)^
Social functioning	50.76 ± 8.88	45.89 ± 9.74	38.98 ± 11.85	<0.001^a(c)^	48.06 ± 9.12	46.84 ± 9.91	38.98 ± 10.90	<0.001^a(d)^
Emotional problems^g^	52.25 ± 4.36	50.06 ± 5.59	48.15 ± 5.59	0.018^a(d)^	50.46 ± 5.38	51.02 ± 5.59	47.55 ± 5.11	<0.001^a(d)^
Mental health	49.92 ± 8.53	48.77 ± 10.08	44.40 ± 10.11	0.049^a(d)^	49.97 ± 8.99	49.92 ± 9.57	43.15 ± 10.27	<0.001^a(d)^

a *K-Wallis test with post hoc comparison Dunn.s test*.

b *ANOVA with post hoc comparison using Bonferroni test; Differences group details (columns 1,2, and 3): (c) 1 > 2; 1 > 3; 2 > 3; (d) 1 > 3; 2 > 3*.

e *Role limitations due to physical problems*.

f *Role limitations due to emotional problems. P < 0.05*.

Table 6 presents the logistic regression models. Teachers who reported no painful regions are significantly associated (*p* < 0.05) with a decreased risk of low scores on the physical (OR: 0.20) and mental QoL components (OR: 0.44). By contrast, individuals with 6 or more painful regions (≥p75) had a greater risk of low scores on the mental (OR: 2.56, *p* < 0.05) and physical components (OR: 2.53, *p* < 0.05). Regarding age, younger teachers (≤44 years) were associated with a significantly lower risk than teachers ≥45 years of having low scores on the Physical Component Summary (OR: 0.45, *p* < 0.01). By contrast, younger teachers had a significantly greater risk than the second age group (≥45 years), of having low scores on the Mental Component Summary (OR: 1.66, *p* < 0.05).

**Table 6:** Logistic regression to evaluate the association between the physical and mental health components of quality of life with the absence of MSD and greater equal to six painful regions (≥p75).

	**PCS (T-Score)**		**MCS (T-Score)**	
	**OR [95% CI]^a^**	***P***	**OR [95% CI]^a^**	***P***
Without MSD	0.20 [0.09–0.45]	<0.01	0.44 [0.24–0.82]	0.01
≥6 regions with MSD	2.56 [1.70–3.86]	<0.01	2.53 [1.64–3.90]	<0.01
Gender (female)	1.38 [0.92–2.05]	0.115	1.27 [0.86–1.88]	0.237
Age (≤44)	0.45 [0.30–0.66]	<0.01	1.66 [1.15–2.42]	<0.01
Hosmer-Lemeshow test^b^	0.748		0.489

*PCS, Physical Component Summary; MCS, Mental Component Summary, MSD, musculoskeletal disorders*.

a *OR, Odds Ratios [Confidence interval]*.

b *A value above 0.05 indicates that the model fits the data; T-Score, 50 indicate good QoL perception while scores below 50 indicate poor QoL perception*.

## Discussion

The objective of this cross-sectional study was to evaluate the association of MSD with QoL in Chilean teachers. The principal results show high MSD prevalence among participants (90.81%). In this regard, teachers with more painful regions had significantly lower scores in all QoL scales, with risks of low scores on both Physical and Mental QoL scales. Age and gender according to their categories were reported as risk factors, where young teachers had a significant risk of low scores on the Mental Component Summary, while teachers in the second age category had a significant risk of low scores on the Physical Component Summary.

### MSD Prevalence

Regarding MSD, 90.81% of teachers in this study reported having felt pain some-where in their body in the previous 12 months, similar to Italian teachers' reported prevalence of 91.5% (47). This MSD prevalence is relatively high considering that the maximum found in literature review about MSD among teachers was 95% prevalence in a Chinese study (23, 48). High MSD prevalence have also been reported among Latin American teachers. For instance, in Bolivia 86% reported pain in at least one part of their body in the previous 12 months (26); other studies in Brazil reported that 55% of teachers mentioned pain in at least one body segment, as well as in Chile where 88.9% of teachers reported MSD in a body region (35, 49).

Regarding MSD in each body region, lower back pain is the most common (80.85%) followed by the neck (47.17%) and knees (45.19%). These regions have already had high prevalence reported among teachers worldwide (50). For example, in Saudi Arabia 81% reported lower back pain, 58% in the neck and 62% in the knees (51); while for teachers in Turkey the prevalence were lower back 74.9%, neck 47.9% and knees 30.9% (36). In this sense, studies among teachers reveal that lower back pain is significantly associated to job provisions, work environment satisfaction (52) and the female gender (53). Additionally, a study during the health crisis by COVID-19 indicates that more than 60% of teachers have some back pain associated with non-performance of PA, non-compliance with ergonomic recommendations, and stress (54). In that vein, the highest pain associations have been reported with extremely severe depression, extremely severe anxiety and high psycho-social work demands among secondary teachers (28). In this regard, this study did not evaluate depressive states. However, teachers with higher MSD prevalence had lower QoL scores on mental health-related scales.

Regarding MSD according to the age variable, each age group in this study presented the same pain prevalence in at least one part of the body in the previous 12 months. These results have no counterpart in the literature, considering that some studies report no MSD associated with the age variable or with any sociodemographic variable in teachers (55). Other studies report greater MSD prevalence among older teachers (56, 57), while some even specifically indicate that ages 40–49 present the greatest MSD prevalence (58). Regarding gender, in this study the highest prevalence were found among women, which coincides with MSD prevalence reported in most studies done on teachers (24). However, a study in Malaysia reported a higher MSD prevalence among women but without significant associations between both genders (56). This contrasts with a study among Turkish teachers, where more MSD presented among men but without significant gender associations (19).

In Chile, there are reports that 42.6% of the general working population has chronic work-related MSD, with symptoms presenting as risk factors strongly associated with low QoL scores, especially on the physical and mental health components (33). Therefore, the organizational context of teaching work in Chile and the world presents various physical and psychosocial deficiencies resulting in the presence of MSD and a concomitant deterioration in the mental and physical health of people.

### MSD and Quality of Life

The findings of this study indicate that scores on all QoL scales present significant differences among teachers, especially between those who report no MSD and those with six or more painful regions (≥p75). Furthermore, having six or more painful regions (≥p75) was reported as a significant risk associated with low scores on the Physical Component Summary and Mental Component Summary. These results are similar to reports from teachers in Turkey where MSD presence was linked with negative QoL impact, especially on the Physical Component Summary (19) and which indicated that teachers with MSD have significantly lower QoL scores and greater presence of depression (36). Similarly, studies on the working population in the USA report high muscle pain prevalence related to low QoL scores (34); and studies on dockworkers in Brazil showed similar significantly lower scores on at least 5 of the 8 QoL scales on the SF-36 instrument among individuals with MSD (32). In the Chilean general population, people with chronic musculoskeletal pain have reported physical and mental QoL impacts, especially among women (33). Studies on the working population of Chilean teachers report high MSD prevalence in urban and rural teachers with significant QoL impact (35). Some chronic health conditions impacting teachers' QoL have already been reported in Chile, a background reinforcing the fact that teachers are an at-risk group for low QoL scores de-pending on the health disorders they may present (20, 35).

### Limitations

This study has several limitations. First, given the nature of this research as a cross-sectional study, only a moment of the interviewees' lives can be observed, which can influence the answer at the time of the interview and prevent studying cause-effect relations. Second, although a representative sample of teachers was obtained, it does not necessarily represent all teachers. However, it has the strength of covering three zones of Chile (north, center, and south) which helps reach a broader vision of MSD and QoL is-sues among teachers.

## Conclusion

This study determined a high prevalence of MSD among Chilean teachers, with significantly greater prevalence among women than men. We also determined the impact of MSD on teachers' QoL, where younger teachers with high MSD had a greater risk of low mental component QoL scores and older teachers with high MSD had a greater risk of low physical component QoL scores. These findings should be useful for generating policies to protect teachers' physical and mental health in order to protect the people who pro-vide education to the youth of the nation.

## Data Availability Statement

The raw data supporting the conclusions of this article will be made available by the authors, without undue reservation.

## Ethics Statement

The studies involving human participants were reviewed and approved by Ethics Committee at Pontificia Universidad de Valparaíso, Chile (n°BIOEPUCV-H 160-2017). The patients/participants provided their written informed consent to participate in this study.

## Author Contributions

PL designed the study. PL and GV-F performed the measurements and statistical analysis, processed the data, drafted the manuscript, designed the tables, writing review, and editing. All authors discussed the results and commented on the manuscript, contributed to the article, and approved the submitted version.

## Funding

This research was supported by FONDECYT Grant No. 11170716 (FONDECYT-ANID, Chile) and Vicerrectoría de Investigación y Estudios Avanzados of the Pontificia Universidad Católica de Valparaíso through project 039.346/2016–039.460/2021.

## Conflict of Interest

The authors declare that the research was conducted in the absence of any commercial or financial relationships that could be construed as a potential conflict of interest.

## Publisher's Note

All claims expressed in this article are solely those of the authors and do not necessarily represent those of their affiliated organizations, or those of the publisher, the editors and the reviewers. Any product that may be evaluated in this article, or claim that may be made by its manufacturer, is not guaranteed or endorsed by the publisher.
